# Mycotoxin concentrations in rice are affected by chalkiness, grain shape, processing type, and grain origin

**DOI:** 10.1007/s12550-024-00575-w

**Published:** 2024-11-27

**Authors:** Erasmus N. Tang, Sali A. Ndindeng, Geoffrey Onaga, Alejandro Ortega-Beltran, Titilayo D. O. Falade, Rousseau Djouaka, Michael Frei

**Affiliations:** 1 Africa Rice Center (AfricaRice), Agri-Food Systems, Crop-Based System, Farming Systems and Postharvest, M’bé, Bouaké, Côte d’Ivoire; 2 Africa Rice Center (AfricaRice), Genetic Innovations, Plant Pathology and Seed Health, M’bé, Bouaké, Côte d’Ivoire; 3 International Institute of Tropical Agriculture, Ibadan, 200001 Oyo State Nigeria; 4 Institute of Agronomy and Crop Physiology, Justus-Liebig-University, 35392 Giessen, Germany

**Keywords:** Mycotoxin, Rice quality, Processing method, Storage, Commercial samples, Africa

## Abstract

Mycotoxins such as aflatoxins (AFs), fumonisins (FBs), zearalenone (ZEN), and deoxynivalenol (DON) pose a risk to public health due to their carcinogenic potency (AFs and FBs) and anti-nutritional effects. The hazards associated with mycotoxins are accentuated where food management practices, control, and regulatory systems from farm to plate are sub-optimal. Information on the frequency of these mycotoxins in rice commercialized in markets in sub-Sahara Africa (SSA) is limited. The current study examined AF concentrations in 527 rice samples collected from 54 markets in five SSA countries. Grain quality characteristics, processing methods, and origin of samples were contrasted with toxin levels. In total, 72% of the samples had detectable AFs levels (range = 3.0 to 89.8 µg/kg). Forty-seven percent (47%) of the samples had AFs above 4 µg/kg, the European Union maximum level (ML), and were evaluated for cooccurrence with FBs, ZEN, and DON. Total AFs and ZEN cooccurred in 40% of the samples, and 30% of the positive ZEN samples had concentrations above the ML of 75 µg/kg. Total AFs did not co-occur with FBs and DON. Multivariate analysis revealed that length-to-width ratio (*p* < 0.0001), mixed variety for width (*p* = 0.04), and chalkiness (*p* = 0.009) significantly influenced aflatoxin concentrations. Slender grains had higher AFs concentrations than bold and medium grains (*p* < 0.0001). Possible strategies to mitigate mycotoxin contamination in rice include improving grain quality traits and practicing proper drying and hermetic storage before and after milling. These findings provide valuable insights for both domestic and international actors in establishing and strengthening regulations and management systems to mitigate rice mycotoxin contamination.

**Supplementary Information:**

The online version contains supplementary material available at 10.1007/s12550-024-00575-w.

## Introduction

The contamination of foodstuffs with mycotoxins, especially the main staples, poses potential threats to global food safety, food security, and public health. In countries experiencing severe food insecurity, mycotoxin contamination will further disrupt food access, affordability and availability. Additionally, it will hinder nutrient digestion, metabolism and physiological growth (Adeyeye et al. 2022). Rice, a major staple with a vital role in food and nutrition security, is susceptible to mycotoxin contamination. The cultivation, processing, and storage of rice in subtropical and tropical environments with warm and humid conditions favor fungal infection, growth, and mycotoxin secretion (Sales and Yoshizawa 2005; Hawkins et al. 2005; Kumar et al. 2021).

Rice contaminated with mycotoxins such as AFs, FBs, ZEN, DON, and ochratoxin above tolerance thresholds becomes rejected in international trade and formal markets as mycotoxins are associated with liver, kidney, lungs, urinary, and digestive tract tumors and immunosuppression in humans (Mcmillan et al. 2018; Chen et al. 2018; Andrews-Trevino et al. 2019; Ekwomadu et al. 2022). These foodborne mycotoxins also act synergistically with hepatitis infections in adults (Chen et al. 2013) and poor nutritional status in children (Khlangwiset et al. 2011), contributing to a high burden of diseases and stunted growth. This occurs through suppression of the immune system resulting in enormous social and economic losses. Total AF contamination of rice has been reported in all major rice production and consumption regions of the world (Elzupir et al. 2017; Tang et al. 2019; Ali 2019). In Africa, the concentration of aflatoxin B1 (AFB_1_) in rice has been reported to range from 1.5 to 10.0 µg/kg in Cote d’Ivoire (Sangare-Tigori et al. 2006), 4.1 to 309.0 µg/kg in Nigeria (Makun et al. 2011), and up to 11.0 µg/kg in Egypt (Madbouly et al. 2012). Rice contamination by AFs has been attributed to warm and humid conditions, poor management practices, mechanical damage during handling (Magan and Aldred 2007), type of fungal strain (Santos et al. 2022), storage temperature, relative humidity, and storage duration (Savi et al. 2018; Al-Zoreky and Saleh 2019; Tang et al. 2019).

Rice is an essential commodity in international trade, and most countries in sub-Saharan Africa (SSA) are net importers (World Bank 2013; USDA 2019; Arouna et al. 2021). Most studies on commercial rice in markets in SSA focused on grain quality (Graham-Acquaah et al. 2019) in relation to consumer preference (Fiamohe et al. 2018, Rutsaert et al. 2013; Demont 2013; Demont et al. 2013a, b; Akoa et al. 2016; Zossou et al. 2022) and effects of grain quality characteristics on rice prices (Peterson-Wilhelm et al. 2021; Ndindeng et al. 2021; Twine et al. 2022, 2023). Few studies reporting on mycotoxin frequency were in-country focused (Sangare-Tigori et al. 2006; Makun et al. 2011; Madbouly et al. 2012), with little attention to regulation or compliance with international standards, despite the huge rice import–export flows between countries in the region. Very few studies have investigated the role of intrinsic characteristics of rice grain quality, processing type and origin of grains on aflatoxin contamination in rice. Tang et al. (2019) showed that the concentrations of AFs, ZEN, and FBs in rice collected from mills in three different climatic zones of SSA were influenced by broken fractions, chalkiness, and impurities. Akbar et al. (2024) examined the effects of grain quality, processing, and production zone on aflatoxin contamination and found that brown rice had the highest aflatoxin levels while protein content was negatively correlated with aflatoxin content.

In the current study, our first objective was to investigate the frequency of AFs in commercial rice samples from markets in SSA and to model in-depth the effects of intrinsic grain quality characteristics such as chalkiness, grain shape, head rice ratio, mixed variety for width (varietal purity), viscosity parameters, amylose content, protein content, type of processing, and origin of rice sample on aflatoxin. Our second objective was to investigate the cooccurrence of FBs, ZEN, and DON with total AFs in samples with high aflatoxin concentration. Results from the current study are expected to improve the knowledge of rice stakeholders on the frequency of mycotoxin in market samples from SSA, conditions influencing mycotoxin accumulation in those samples, and the strategies to be adopted to reduce the risks associated with the consumption and trade of mycotoxin contaminated rice.

## Materials and methods

### Sample collection

The samples analyzed in this study were collected from Cote d’Ivoire, Kenya, Madagascar, Nigeria, and Uganda. A sample collection protocol was developed and implemented to consistently record sample information (origin—domestic or imported, type of processing—brown, parboiled, or white, and type of market—rural or urban) and to ensure that the samples were representative of the rice consumed in each country. The main rice production and consumption regions were identified in each country. Regions were randomly selected for sample collection using the stratified sampling technique. In each region, rural and urban rice markets were listed, and simple random sampling was used to select the markets for sample collection. Urban areas are characterized by a continuum of markets from low to high end. In each market, focus group discussions were organized with market leaders to explain the objective of the study and to identify rice retailers. In each market, rice retailers were randomly selected from the identified list using the simple random sampling method in XLSTAT (Addinsoft 2023). At each retailer level, the objectives of the study were explained and the retailers that were selected were those who voluntarily accepted to participate in the study. Rice samples of at least 0.25 kg were purchased from each retailer. Only one sample per branded rice type was purchased from each selected retailer with a maximum of five samples purchased for each market. Unbranded samples at each retailer level were purchased since such samples could not be effectively linked to previously purchased samples from other retailers in that market. Where a retailer sold more than one type of rice, the samples to be purchased were randomly selected. Samples were collected and immediately stored in sterile hermetically sealed aluminum foil bags and shipped to the Grain Quality and Post-harvest Laboratory at Mbe, Bouake, Cote d’Ivoire for grain quality and mycotoxin analysis. Once at the laboratory, samples were thoroughly cleaned to remove all foreign matter (stones, sand, dust, and plant material), 25 g of whole grains ground to fine flour through a 0.5-mm mesh sieve using a cyclone mill (UDY cyclone mill; Fort Collins, CO, USA) and stored at < 4 °C prior to analysis.

### Physicochemical analysis of rice samples

Routine grain quality analysis was performed on all samples. The milled head rice ratio was calculated as the percent weight of head rice (proportion of intact grains or grains with 75% intact length) relative to 100 g of the collected sample. The grain dimensions (length and width) were determined in 50 g of whole grain sample using the S21 Rice Statistical Analyzer (LKL Tecnologia Ltda, Brazil) as described by Graham-Acquaah et al. (2019). The length-to-width ratio (LWR) which defines grain shape classified as bold (LWR < 1), medium (1 > LWR < 2.9), and slender (LWR > 3); mixed variety for width (varietal purity), which is the percentage of grains with width different from 95% of the sample were retrieved from the S21 output results. Chalkiness, which is the percentage of chalky grains (grains with a white belly) relative to the total chalky area was classified as chalky (percentage of grain with chalky area > 20%) and nonchalky (percentage of grain with chalky area < 20%) were determined in a 50 g sample using the S21 Rice Statistical analyzer (LKL Tecnologia Ltda, Brazil) as previously described (Ndindeng et al. 2021).

The grain color was measured with a colorimeter (CR-400, Minolta Co., Ltd., Tokyo, Japan) and expressed as Hunter Lab (lightness (L), red (a), and yellow (b)) values (Zohoun et al. 2018). Amylose and protein content were determined using a pre-calibrated near-infrared spectroscopy equipment (AN820 Rice Composition Analyzer, Kett, CA, USA). Flour was produced by grinding samples in a cyclone mill (UDY cyclone mill; Fort Collins, CO, USA). The flour pasting properties (peak, breakdown, final, and setback viscosities) were determined using the Rapid Visco Analyzer model- super4 (Newport Scientific, Warriewood, Australia) as previously described (Ndindeng et al. 2015). The gelatinization temperature (GT) was determined using the Dang and Bason (2014) method with slight modifications. Briefly, a concentrated rice flour slurry (6.00 ± 0.01 g, corrected to 12% moisture basis) in 24.0 ± 0.1 g of distilled water was heated at 50 °C for 5 min, then from 50 to 95 °C at 3 °C/min while recording the viscosity of the sample throughout. GT, observed at the point of rapid increase in viscosity, is defined as the temperature at which the viscosity first increases by at least 24 centipoises (cP) in 6 s. The test was manually stopped when the viscosity reached 4100 cP, which occurred between 11 and 20 min of the test.

### Mycotoxin analysis

#### Extraction of total aflatoxin, fumonisins, zearalenone, and deoxynivalenol

Mycotoxin extraction was carried out following the Neogen Reveal Q + kit procedure (Le et al. 2019; Gray et al. 2020) with slight modifications. For the extraction of total aflatoxin, 5 g flour was weighed in a recipient and 25 mL 65% ethanol was added. The mixture was vigorously homogenized for 3 min by manual shaking and allowed to settle. Subsequently, 3 mL of the supernatant was transferred to an Eppendorf tube and centrifuged at 5100 rpm for 5 min. A 100 µL aliquot of the extract was transferred to the red sample cups of the Reveal Q + kit for aflatoxin, followed by the addition of 500 µL of sample diluent. The aliquot and the sample diluent mixture represent the extract that is set for the quantification of total aflatoxin by the raptor reader (Neogen Corp., Lansing, MI).

Samples with total AFs ≥ 3 µg/kg (lowest detectable levels of the quantification method used) were purposefully sampled (50 samples each) to verify the cooccurrence of total FBs, ZEN, and DON with total AFs. To this effect, total FBs was extracted following the same procedure for total AFs. ZEN and DON were extracted in 5 g of the sample by vigorously homogenizing with the corresponding 1 MAX powder pack supplied with the kits. The resulting homogenate was mixed for 1 min in 25 mL of deionized water and allowed to settle. The supernatant (3 mL) was centrifuged at 5100 rpm for 5 min and a 100-µL aliquot was diluted 10 times with the sample diluent and gently homogenized before quantification.

#### Quantification of total aflatoxin, fumonisins, zearalenone, and deoxynivalenol concentrations

The quantification of total AFs, FBs, ZEN, and DON was realized using the Neogen Reveal Q + kits. The operation procedure for mycotoxin quantification was based on the principle of competitive enzyme‐linked immunosorbent assay (ELISA) plate kit (Beacon Analytical Systems, Inc. Saco, ME) methods (Le et al. 2019; Gray et al. 2020). After calibration of the raptor reader, the sample strip from the AFs kit (or FBs, ZEN, or DON) was inserted into a unique cartridge, and the QR code was scanned to enter the sample lot ID into the scanner followed by the specific sample code. Subsequently, 400 µL of the prepared extract was slowly pipetted into the opening of the cartridge funnel and allowed to read for 6 min (AFs and FBs), 5 min (for ZEA), and 3 min (for DON). Lateral flow strips coated with antibodies interact with antigen (mycotoxin) molecules from the extract during the incubation period, and the antigen–antibody binding capacity is read as the given mycotoxin concentration by the scanner. The linear range of AFs detection is 3–100 µg/kg, ZEN 50–500 µg/kg, and FBs and DON 0.3–6 mg/kg. Sample readings that exceeded the upper limits were diluted and mycotoxin concentration was calculated taking into account the dilution factor. For total aflatoxin dilution, 100 µL of the extract was transferred to the sample collection tube and 100 µL 65% ethanol was added and homogenized. Then 100 µL of the dilution was pipetted and mixed with 500 µL of the sample diluent in the red sample cup, followed by a reading of 400 µL in the reader.

### Statistical analysis

Each grain quality analysis was performed in three replicates, while all samples in Table 1 were analyzed for total aflatoxin. To model the quantitative variables explaining aflatoxin concentration in rice, head rice ratio, LWR, chalkiness, final viscosity, mixed variety for width, protein content, amylose content, and impurities as the dependent variables and aflatoxin as the independent variable were run in a stepwise variable selection regression process. The model options were fixed intercept, 95% confidence interval, the probability for entry was 0.05, probability for removal was 0.1, and tolerance level was 0.0001. For qualitative variables, a multivariate model with 5 factors, namely; rice origin (2 levels—domestic and imported (benchmark)), processing type (3 levels—brown, parboiled, and white (benchmark)), type of market (2 levels—rural and urban (benchmark)), grain shape classification (3 levels—bold, medium, and slender(benchmark)), and chalky grain classification (2 levels—chalky and nonchalky (benchmark)) was used to verify their effects on aflatoxin concentration. Given the significant effects of length-to-width ratio and chalkiness on total aflatoxin concentrations, the data were further segregated by these factors, and the mean and range were used to assess the level of aflatoxin in each group. All models were run at *p* < 0.05 and at a tolerance level of 0.0001. All analyses were performed in XLSTAT 2023, version 1.2.

**Table 1:** Summary frequency of the collected rice samples as a function of origin, processing, type of market and grain classification by grain shape and chalkiness

Variable	Category	Counts	percentage
Origin	Domestic	257	48.77
	Imported	270	51.23
Processing	Brown	21	3.99
	Parboiled	61	11.58
	White	445	84.44
Type market	Rural	118	22.39
	Urban	409	77.61
Grain shape classification	Bold	23	4.36
	Medium	355	67.36
	Slender	149	28.27
Chalky grain classification	Chalky	198	37.57
	Not chalky	329	62.43

Grain shape classification (LWR < 2.1 = bold; LWR 2.2–2.9 = medium; LWR > 3 = slender), chalky grain classification (< 20% = not chalky; > 20% = chalky)

*LWR* length-to-width ratio

## Results and discussions

### Description of the samples

A total of 527 samples were collected from Cote d’Ivoire, Kenya, Madagascar, Nigeria, and Uganda. The summary statistics of the samples are shown in Table 1. Fifty-one percent (51%) of the samples were imported confirming the dependence of SSA on rice imports to meet local demands (USDA 2019; Arouna et al. 2021). Eighty-four percent (84%) of samples were straight milled polished (white) rice, 12% were parboiled polished rice, and the rest were straight milled nonpolished rice (brown). Consumer studies have shown that the evolution in rice milling systems from simple to more complex multi-stage mills has exposed consumers to white rice, and this has increased preference towards white rice compared to other types (parboiled and brown). However, it is worth noting that this pattern varies within countries and between countries. For examples, about 95% of the samples from Nigeria were parboiled while 98% from Uganda and Kenya were white (nonparboiled). Seventy-eight percent (78%) of the samples were purchased from urban markets while the rest came from rural markets. Urbanization has been linked to increasing rice consumption because it is easy to cook, thereby making urban centers suitable for a diversity of rice brands. About 67% of the samples were medium, 28% slender, and 5% bold grains. In addition, 62% were non chalky while the rest were chalky.

### Physicochemical characteristics of rice

Physicochemical characteristics of rice from rural and urban markets of SSA are shown in Table 2. The head rice ratio (HRR) was higher (*p* < 0.0001) in imported (85%) than in domestic samples (79%). HRR was also higher (*p* < 0.0001) in parboiled samples (94%) than in white (79%) and brown (73%) samples. HRR in rural market samples (81%) was similar to that of urban markets (83%) (*p* > 0.05). Domestic rice samples had lower HRR due to the use of sub-optimal on-farm and postharvest practices (Mapiemfu et al. 2017; Ndindeng et al. 2021). HRR is higher in parboiled samples because parboiling has been shown to heal pre-existing fissures in rice grains resulting in less breakages during milling (Ndindeng et al. 2015). Rice samples sold in rural and urban markets had high (81 and 83%) and comparable HRR, confirming earlier observations that rice consumers in SSA prefer rice with intact grains (Ndindeng et al. 2021).

**Table 2:** Physicochemical properties of rice samples from sub-Saharan Africa by origin, type of processing and markets

Category	HRR (g)	LWR	MVW (%)	Chalkiness (%)	Color intensity	Protein content (% db)	Amylose content (%)	PV (cP)	BDV (cP)	FV (cP)	SBV (cP)	impurities (%)
**Origin of samples**												
Domestic	79.43^b^	2.88^a^	25.63^a^	9.00^a^	9.28^a^	9.75^a^	17.32^a^	1726.28^a^	388.78^a^	3002.87^a^	1277.27^a^	2.630^a^
Imported	85.05^a^	2.92^a^	19.80^b^	10.83^a^	9.05^a^	9.66^a^	17.44^a^	1453.90^b^	260.96^b^	2556.630^b^	1102.00^b^	2.193^a^
**Type of processing**												
White	79.07^b^	2.75^b^	25.10^b^	19.12^a^	8.51^b^	9.62^b^	17.75^a^	2317.92^a^	536.31^a^	3814.15^a^	1495.66^a^	1.743^b^
Brown	73.35^b^	2.61^b^	36.54^a^	9.10^b^	9.00^b^	10.87^a^	17.68^ab^	1915.57^b^	385.34^a^	3622.97^a^	1707.29^a^	5.069^a^
Parboiled	94.30^a^	3.34^a^	6.50^c^	1.53^b^	9.99^a^	8.64^c^	16.71^b^	536.79^c^	52.95^b^	902.14^b^	365.96^b^	0.422^c^
**Type of market**												
Rural	81.22^a^	2.90^a^	24.48^a^	8.24^b^	9.30^a^	9.79^a^	17.45^a^	1638.84^a^	350.14^a^	2809.26^a^	1170.78^a^	2.520^a^
Urban	83.25^a^	2.90^a^	20.94^a^	11.59^a^	9.03^a^	9.62^a^	17.30^a^	1541.33^a^	299.59^a^	2750.25^a^	1208.49^a^	2.303^a^

Values in the same column for each group of categories with different superscript letters are significantly different at *p* < 0.05 level of significance using Tukey HSD test

*HRR* head-to-rice ratio, *LWR* length-to-width ratio, *MVW* mixed variety for width, *PV* peak viscosity, *BDV* breakdown viscosity, *FV* final viscosity, *SBV* setback viscosity Color: red > yellow > green

The LWR of samples from the domestic market was 2.88 and 2.92 for imported samples. For the type of processing, white (2.75) and brown (2.61) rice samples had comparable LWR while that of parboiled (3.34) samples was higher (*p* < 0.05). The average LWR of samples from the rural and urban markets was 2.90. The samples from the current study reveal that most rice in SSA falls in the medium shape classification for brown and white rice and in the slender shape for parboiled rice. In an earlier study, Twine et al. (2022) reported that consumers in SSA manifested significant preference for slender, intact grains, parboiled rice while rice sold in urban markets with slenderness attracted the highest price premium of 45 $/kg. Mixed variety for width, which is an indication of varietal purity, showed a wide distribution between the samples with higher values in domestic (25.6%) than imported (19.8%) samples (*p* < 0.05). This is an indication that varietal purity of domestic samples is lower than that of imported samples and may reflect the practices of mixing rice varieties during paddy aggregation. In the rice type group, brown (36%) samples had the highest percentage of mixed variety for width compared to white (25%) and parboiled (6%). Meanwhile, samples from the rural (24%) and urban (20%) markets showed comparable mixed variety for width values. Chalkiness was 10% in imported and 9% in domestic samples. In the rice type group, parboiled rice showed the lowest chalkiness (1%) compared to white (19%) and brown (9%). However, the average chalky percentages indicate that all the samples, irrespective of processing type felt in the nonchalky (chalkiness < 20%) class. The samples from urban (11%) and rural (8%) markets were also nonchalky. Earlier studies on commercial rice quality from SSA found that chalkiness ranged from 2.1 to 22.0%, respectively, in samples from Nigeria and Cote d’Ivoire. It is known that chalkiness lowers the market value of rice (Ndindeng et al. 2021) as it adversely affects the milling, appearance, cooking qualities and the palatability of cooked rice (Cheng et al. 2005; Xi et al. 2021). Color intensity was comparable between domestic and imported samples and between samples collected from rural and urban markets. In the type of processing category, color intensity was significantly (*p* < 0.05) higher in parboiled samples than in brown and white samples. In parboiled rice, the high color intensity is due to the migration of colorful compounds from the bran layer into the endosperm during soaking and enzymatic browning during steaming (Lamberts et al. 2006). The presence of colorful bioactive compounds such as anthocyanidins and other antioxidants in brown and parboiled rice is responsible for the high color intensity in those samples (Colombo et al. 2023).

The protein content (on a dry matter basis) was 9% in domestic and imported samples and in samples collected from rural and urban markets. For the processing type, brown samples had the highest (11%) protein content, and this was significantly (*p* < 0.05) different from that in white (9%) and parboiled (8%). Sun et al. (2008) reported a protein range of 5–13 g /100 g in rice collected from the major production zones in China while Derycke et al. (2005) reported a protein range of 6–10% in nonparboiled rice and 7–10% in parboiled rice of the same cultivars from Europe and Asia. The amylose content (17%) of all the rice samples was comparable across the different categories; origin of samples, type of processing, and market, indicating that the samples felt within the low amylose class (12 < amylose < 20%), corroborating the apparent amylose content of previous market samples from SSA (Ndindeng et al. 2021; Twine et al. 2023). The viscosity parameters showed high variability, with overall peak, breakdown, final, and setback viscosities higher in domestic than in imported samples. Peak, breakdown, and final viscosities were highest in white rice compared to brown and parboiled samples and highest in samples collected from rural markets compared to those from urbans markets. Setback viscosity was highest in brown samples compared to parboiled rice and was comparable in samples from rural and urban markets. Zohoun et al. (2018) reported lower viscosity values for parboiled rice, revealing that viscosity decreases with increasing steaming time. The level of impurities was the same (2%) in domestic and imported samples and in samples from rural and urban markets. In the rice type category, brown rice recorded the highest level of impurities (5%) than white (1%) and parboiled (0.4%). Higher levels of impurities in rice reduce its quality and negatively affects consumer perception and pricing (Akoa-Etoa et al. 2016; Ndindeng et al. 2021).

### Modeling the effects of grain quality characteristics on aflatoxin concentration in rice

The concentration of total AFs in domestic and imported rice in relation to grain shape and chalky status classifications is shown as supplementary information (SS1). From the stepwise regression model, three dependent variables, viz; LWR (*p* < 0.0001), mixed variety for width (*p* = 0.04), and chalkiness (*p* = 0.009), explained the AF concentration in imported and domestic rice commercialized in SSA (Table 3). This model could not however be used for predicting AFs due to its low predictive power (*R*^2^ = 0.352). The model revealed that AFs increased with an increase in LWR, chalkiness, and mixed variety for width. This implies that grains with low LWR values (tend to be bold), nonchalky area (nonchalky), and mixed variety for width (tend to be pure and homogenous) are less likely to be contaminated with AFs, while those with high LWR (tend to be slender), chalky grains, and mixed variety for width values (tend to be impure and heterogenous) are more likely to be contaminated.

**Table 3:** Model effects of some quantitative physical grain attributes on aflatoxin concentration in rice from rural and urban markets of sub-Saharan Africa

Source	Value	Standard error	*t*	Pr >|*t*|	Lower bound (95%)	Upper bound (95%)	*p*-values signification codes
Intercept	0.000						
Milled head rice (g)	0.000	0.000					
LWR	1.065	0.174	6.119	< 0.0001	0.723	1.408	***
Mixed variety for width (%)	0.025	0.012	2.054	0.041	0.001	0.050	*
Color damage (%)	0.000	0.000					
Chalkiness (%)	0.046	0.017	2.615	0.009	0.011	0.080	**
Color intensity	0.000	0.000					
Protein content (% d.b)	0.000	0.000					
Amylose content (%)	0.000	0.000					
Final viscosity (cP)	0.000	0.000					
Impurities (%)	0.000	0.000					

Signification codes: 0 < *** < 0.001 < ** < 0.01 < * < 0.05 < ns < 0.1 < ns < 1, * = significant, ns = not significant

The grain shape and chalky classifications were further used in a second (qualitative) model to verify this trend, and the results are reported in Table 4. The stepwise model excluded milled HRR, damaged grains, color intensity, protein content, amylose content, final viscosity, and impurities. Therefore, the excluded parameters did not significantly correlate with AFs. Akbar et al. (2024) reported that protein content negatively correlated with AFs in parboiled rice. In contrast, findings of the current study showed that protein content does not significantly influence total AFs in rice. The model also excluded impurities, contradicting Akbar et al. (2024) and Tang et al. (2019), who showed that foreign matter positively influenced aflatoxin concentration in white and parboiled rice. Our model excluded the amylose content and the final viscosity, but a positive correlation has been established between the amylopectin content and the accumulation of AFB_1_ in rice, while a negative correlation with the amylose content has been established (Singh and Sinha 2013). High amylose rice tends to have a high final viscosity (Pang et al. 2016; Shi et al. 2022). Furthermore, the *Waxy* gene (*Wx*) locus in rice has been found to control both the chalky trait and most viscosity parameters (Zheng et al. 2012). From these observations, the relationship between final viscosity and AFs in rice appears to be controlled by multiple complex factors and warrants further investigation. This relationship is important in mitigating or reducing the concentration of mycotoxin in gelatinized starch products, such as parboiled or cooked rice. We found that chalkiness and LWR positively and significantly influenced AFs concentration in market samples. The results on grain chalkiness corroborate with Tang et al. (2019), who suggested that chalky grains demonstrate poor starch formation, facilitating fungal invasion and mycotoxin secretion. Akbar et al. (2024) found that total AFs was weakly related to chalky grains in parboiled rice. The LWR results suggested that grains with high LWR values tend to accumulate high concentrations of AFs, and this may be due to the high specific surface area for microbial growth in slender grains than in the other grain shape types. However, this requires further investigation.

**Table 4:** Model effects of qualitative factors on aflatoxin concentration in rice from rural and urban markets of sub-Saharan Africa

Source	Value	Standard error	*t*	Pr >|*t*|	Lower bound (95%)	Upper bound (95%)	*p*-values signification codes
Intercept	0.000						
Origin code-domestic	8.348	0.487	17.128	** < 0.0001**	7.391	9.306	***
Origin code-imported	0.000	0.000					
Processing code-brown	− 3.182	1.305	− 2.439	**0.015**	− 5.746	− 0.619	*
Processing code-parboiled	− 1.377	0.766	− 1.798	0.073	− 2.882	0.128	ns
Processing code-white	0.000	0.000					
Type market-rural	− 0.956	0.595	− 1.607	0.109	− 2.126	0.213	ns
Type market-urban	0.000	0.000					
Grain shape classification-bold	− 5.040	1.248	− 4.037	** < 0.0001**	− 7.493	− 2.587	***
Grain shape classification-medium	− 3.957	0.424	− 9.333	** < 0.0001**	− 4.789	− 3.124	***
Grain shape classification-slender	0.000	0.000					
Chalky grain classification-chalky	− 0.331	0.496	− 0.668	0.505	− 1.306	0.643	ns
Chalky grain classification-not chalky	0.000	0.000					

Signification codes: 0 < *** < 0.001 < ** < 0.01 < * < 0.05 < ns < 0.1 < ns < 1, * = significant, ns = not significant

To investigate the effects of rice origin, type of processing, type of market, grain shape, and chalky classifications on AF concentration, a multivariate model with imported, white, urban, slender, and nonchalky as benchmark factors was used (Table 4). The model revealed that domestic rice tends to accumulate significantly (*p* < 0.0001) more AFs compared to imported rice. This could be an indication that the supply chain management practices, quality control, regulation measures, and infrastructure used in the domestic rice value chain are sub-optimal and not adequate in preventing fungal invasion and AF secretion. This result is in line with Hassan et al. (2022), who found that the packaging country and country of origin did not affect the accumulation of AFB_1_ in imported rice marketed in Lebanon. Imported white and parboiled rice in most of SSA countries comes from Asia, United States of America, and Brazil, and it was, however, previously reported that mycotoxin frequency in rice from these regions increased steadily between 2005 and 2011 (Streit et al. 2013). This is an indication that the mycotoxin control and regulation systems in countries exporting rice to Africa may also be weak, justifying AF contamination in imported rice detected in this study.

Brown rice had less AFs compared to parboiled and white rice (*p* = 0.015). Although AFs concentration was slightly higher in white compared to parboiled rice, this was not significant (*p* > 0.05). Almeida et al. (2012) and Hassan et al. (2022) reported that brown rice for the same variety expressed higher AFs, with Almeida et al. (2012) revealing that the milling process reduced AFs levels from brown rice to white rice by almost 78%, when compared to its by-product (bran). Furthermore, Trucksess et al. (2011) also showed that for the same variety stored on a farm under humid and poorly ventilated conditions, the bran layer and brown rice had the highest levels of the sums of AFB_1_ and aflatoxin B2. Bran layer on brown rice may harbor more fungal spores and accelerate their proliferation given its high nutrient density, fiber, and natural oils. Oils can quickly become rancid when stored improperly in warm and humid conditions to attract more fungal spores. However, in the current study, white rice had the highest AFs level than brown and parboiled rice. Two reasons may explain this observation; most likely, the small sample size of brown rice (4% vs. 84% for white rice) as it is not common in the markets nor commonly consumed in SSA and the fact that the samples were independent of each other (not of the same variety) and might have undergone different handling operations that may have exposed white rice to conditions leading to fungal invasion and mycotoxin accumulation. Parboiled rice had slightly lower levels of AFs than white rice, in agreement with Trucksess et al. (2011), Almeida et al. (2012), and Hassan et al. (2022). Low concentration of mycotoxins in parboiled rice is believed to be linked to several mechanisms initiated by hydrothermal operations and chemical changes that occur during parboiling (Dors et al. 2011). The hydrothermal soaking and steaming and starch gelatinization make parboiled grains drier and harder, making them less hospitable to fungal spores. Long-term hot soaking facilitates the leaching of water-soluble mycotoxins (citrinin, ochratoxin A, patulin, Alternaria toxins, and penicillic acid) and high temperature steaming (> 100 °C) induces the degradation of heat-labile mycotoxins (AFB_1_ and DON), contributing to the reduction of mycotoxins in parboiled rice.

### Cooccurrence of aflatoxin with fumonisins, zearalenone, and deoxynivalenol in rice

The results for the cooccurrence of AFs with FBs, ZEN, and DON together with the compliance checks against the ML of the EU (European Food Safety Authority 2013, 2014; European commission 2023) are shown in Table 5. About 72% of samples had detectable levels for total AFs (3.0–89.8 µg/kg) of which 47.5% exceeded the EU ML. Total AFs cooccurred with ZEN but not with FBs and DON. ZEN was detectable in 40% of the samples at levels of 25.7 to 596.7 µg/kg. Sixty percent (60%) of the samples presented levels of ZEN below the LOD (limit of detection), thus considered not contaminated. FBs (0.00–0.09 mg/kg) and DON (0.00–0.13 mg/kg) occurred at concentrations below the LOD (quantification range was 0.30–6.00 mg/kg for the two toxins) and therefore, none of the samples was considered contaminated by these toxins. Multiple mycotoxins have been reported in rice from Kenya (Mutiga et al. 2021), Nigeria (Makun et al. 2011), Benin, Cameroon, Senegal (Tang et al. 2019), Brazil (Almeida et al. 2012), Cote d’Ivoire (Manizan et al. 2018), China (Sun et al. 2017), and Pakistan (Majeed et al. 2018). Makun et al. (2011) reported the cooccurrence of five mycotoxins; AFs, ZEN, FBs, DON, and ochratoxin A in four different combinations (2, 3, 4, and 5) in field, stored, and market rice from traditional production areas in Nigeria. It has been established in animal models that multiple mycotoxin cooccurrence in feed exerted a synergistic effect on toxicity. Huang et al. (2018) showed that a combination of AFB_1_, ochratoxin A, and ZEN in lactating dairy goats significantly increased alanine aminotransferase and alkaline phosphatase activities, total bilirubin, interleukin-6, and malondialdehyde than in the group fed one of the toxins or no toxin. The same treatment significantly reduced immunoglobulin A concentration, the activities of superoxide dismutase and glutathione peroxides (GSH-Px), and total antioxidant capacity (T-AOC) in serum (Huang et al. 2018). While investigating the cytotoxic effects of mycotoxin in rats, Sun et al. (2015) found that the mixtures of AFB_1_ plus ZEN and AFB_1_ plus DON exerted synergetic toxic effects in BRL 3A liver cells.

**Table 5:** Mycotoxin contamination of rice from sub-Saharan African markets

Toxin	No. samples	Range	No. samples with mycotoxin occurrence	ML*	No. samples considered contaminated (> ML)
Aflatoxin (µg/kg)	527	3.00–89.8	379	4	180
Fumonisin (mg/kg)	50	0–0.09	0	1	0
Zearalenone (µg/kg)	50	25.7–596.7	20	75	6
Deoxynivalenol (mg/kg)	50	0–0.13	0	0.75	0

^*^ML according to the European Union (European Commission 2023; European Food Safety Authority 2013, 2014)

*ML* maximum level, *µg/kg* parts per billion, *mg/kg* parts per million

Regarding the compliance with international safety regulations and standards, total AFs occurrence was found in 71.9% of the samples, and 47.5% of the samples had AFs above the ML (4 µg/kg–EU standard), thus contaminated. For ZEN, 30% of the samples exceeded the ML of 75 µg/kg. Mycotoxin control and regulation in African countries are weak, and most countries rely on the standards of the EU for quality control and trade. Of the five countries considered in the current study, three had established and updated their mycotoxin regulation, but none of the regulations was specific to rice or grains intended for direct human consumption (Van Egmond and Jonker 2005). For example, the regulation in Cote d’Ivoire is focused only on total AFs and feedstuffs, with the maximum level for total AFs set at 100 µg/kg and 10 µg/kg for direct feedstuffs and complete feedstuffs, respectively (Van Egmond and Jonker 2005; FAO 2004). In Kenya, MLs are established for all foods (AFB_1_; 5 µg/kg), milk and milk products (aflatoxin M1; 0.05 µg/kg) and 20 µg/kg for total AFs in peanuts, products and vegetable oils (Van Egmond and Jonker 2005; Senerwa et al. 2016; East African Standards (EAS) 2011; Ankwasa et al. 2021). The high rates of total aflatoxin and the incidence of cooccurrence reported in the current study add to previous similar reports. underlining the need for African countries (at individual or regional level) to establish, strengthen, and operationalize mycotoxin regulations to ensure safe food access to the populations.

### Strategies to mitigate the risk of mycotoxin contamination in rice

A greater proportion of rice cultivation, processing, and commercialization (including the retail of imported rice to the final consumer) in SSA is in the hands of small-scale actors and the management system is characterized by inadequate technologies during pre-harvest, harvest (Mapiemfu et al. 2017), and postharvest (Ndindeng et al. 2021). Inappropriate technologies and practices combined with characteristic humid and sub-humid tropical temperatures (~ 15.8–34.9 °C) and relative humidity of 34.2–89.7% (Tang et al. 2019), and structures of communities of toxigenic fungi, are the underlying factors that favor mycotoxin contamination of food crops. In addition, farmers face unprecedented extreme climatic events and lack basic knowledge of rice grain quality management and maintenance. These factors add up to explain the high contamination of domestic and imported rice sold in markets with AFs and the incidence of cooccurrence with other mycotoxins. Paddy harvested under humid conditions, processed with a high moisture content (> 14%) and handled (retailed, transported, and stored) under improper, poorly ventilated, nonhermetic, and uncontrolled conditions, promotes fungal infection (Tang et al. 2019) and AF contamination (Trucksess et al. 2011; Douksouna et al. 2019). The optimal temperature and relative humidity suitable for the growth of *Aspergillus* spp. and AF secretion in grains is between the ranges of 25 and 37 °C and 75 and 100% (Hawkins et al. 2005; Siciliano et al. 2017). In modern rice production systems, such as in Japan and Taiwan, mycotoxin contamination has been reported at 0 µg/kg (Chandravarnan et al. 2024) or rarely occurs (Tanaka et al. 2007) thanks to the application of adequate technologies and practices. Therefore, preventing rice from mycotoxin contamination in SSA requires a holistic application of appropriate technologies, policies, and institutional innovations. Practices for mitigating mycotoxin contamination of rice in SSA which are adapted to smallholders in terms of skills, affordability, and accessibility are discussed in the following section.

#### Breeding strategies

Our model results showed that broken grains, chalkiness, and slenderness predispose rice samples to AFs contamination. Therefore, breeding for these attributes will contribute to mycotoxin control in rice. The *chalk5* gene has been identified as contributing to low chalkiness in rice grains (Li et al. 2014). The *chalk5* gene is a vacuolar H-pyrophosphatase, with class B alleles (allele types 5, 6, and 7), conferring lower chalkiness. Additionally, the *qPGC8-2* and *qPGC8-1* quantitative trait loci (QTLs) have also been shown to control grain chalkiness and grain shape on rice chromosome 8 by substitution mapping and their additive effects on grain chalkiness were not affected by higher temperature (Yang et al. 2021). Low chalkiness is attributed to grain firmness and less susceptibility to breakage and fungal infestation. Slenderness was shown in the current study to favor AF accumulation. However, sensory acceptability studies showed that consumers prefer slender grains (Ndindeng et al. 2021; Twine et al. 2022). To prioritize consumer preferred attributes, breeding should continue to select for slender grains while other mycotoxin mitigation methods are applied. Rice varieties that are naturally resistant to fungi and mycotoxin secretion can be selected through breeding and promoted for adoption by farmers. However, rice varieties resistant to fungi are generally not common (Khush and Jena 2009; Deng et al. 2017), especially in SSA. Emerging advanced technologies such as gene editing can be applied to develop rice varieties resistant to fungi.

#### Cropping system practices

Mycotoxin-producing fungi are diverse and can infect rice both in the field and after harvest. The well-known fungal species affecting rice are *Aspergillus* spp. (*A. flavus, A. ochraceus,* and *A. versicolor*), *Fusarium* spp. (*F. armeniacium, F. fujikuroi, F. graminearum, F. proliferatum*, and *F. subglutinans*), Penicillium spp. (*P. aurantiogriseum, P. citreonigrum, P. citrinum, P. commune, P. islandicum, P. rugulosum*, and *P. verrucosum*), Alternaria spp. (*A. infectoria* and *A. tenuissima*), and *Ustilaginoidea virens*. Given this diversity of rice fungal pathogens, rotation with maize, legumes, potato, wheat, or fodder crops is believed to disrupt the growth cycle of fungal spores, which may help in reducing the presence of mycotoxins in grains (Bernhoft et al. 2012). Cropping operations with positive fungal infection control include adequate use of fungicides, fertilization, irrigation, and proper field management to reduce stress on the rice plants during maturity, especially at the grain filling stage. The use of fungicides is sensitive and should be exercised with caution, as some are positively linked to AFs, ochratoxin A, ZEN, and DON contamination in rice (Dors et al. 2013). There is also the emergence of fungicide-resistant pathogen strains and the negative effects of fungicides on soil biodiversity and human health. An option to replace fungicides are the use of bioprotectant agents (such as atoxigenic fungal species) because of their safety and eco-friendliness in the control of mycotoxins in food and feed. In the case of AFs, atoxigenic isolates of *A. flavus* are currently being used in some SSA countries to mitigate AFs contamination of several staple crops (Ortega-Beltran and Bandyopadhyay 2021). However, the use of atoxigenic fungi isolates as biocontrol agents in the mitigation of mycotoxin in rice in SSA has not been investigated and requires attention.

#### Harvest and postharvest strategies

Equipment used for rice harvesting and postharvest operations can be a source for mycotoxigenic fungal contamination (Gummert et al. 2009). Rice handling involves the use of sickles, bags, and transportation equipment. Drying equipment includes tarpaulins, constructed drying pavements, bare floors, potable moisture meters (for the monitoring of grain moisture content), and rakes for stirring the grains. Using clean equipment, uncontaminated with soil that might contain fungal spores, reduces the risk of mycotoxin contamination. In addition to keeping handling equipment clean, it is recommended to reduce rice overhandling, as this increases the number of cracked and damaged grains, which are highly prone to fungal invasion (Gummert et al. 2009).

Drying the paddy immediately after harvest to the recommended moisture level (14% in many countries) and avoiding rehydration before milling is important to maintain quality. The thermodynamic water activity (*a*_w_) of rice after harvest and milling is the key determinant of the growth of storage fungal spores. High water activity of rice grains causes fungal growth, and this occurs at *a*_w_ above 0.85, equivalent to equilibrium relative humidity (ERH) above 85% (Abdel-Hadi et al. 2011). Adequate drying of paddy after harvest to below 65% ERH (i.e., below 14% MC) reduces the metabolic activity of microorganisms and insects, preventing the growth of fungal spores and the accumulation of mycotoxin during storage. Proper drying of rice equally minimizes grain damage in terms of crack formation and breakage during milling, thus preventing fungal infestation. In SSA, the sun is the main source of energy for drying rice; therefore, paddy drying should be avoided on rainy days. To achieve drying efficiency (high milling recovery and high head rice yield), it is recommended that paddy be harvested around 25 (± 5) days after 75% flowering and immediately sun dried (dry chain) only in the morning, not throughout the day (Xangsayasane et al. 2018). Mechanical dryers and other hot air-drying techniques, such as flat-bed dryers, although rare, should be promoted for efficient rice drying.

The mature paddy should be harvested, threshed and milled with appropriate equipment to minimize grain breakage. Rice is often threshed by hitting panicles on a stone, wood, or metal drum placed on a tarpaulin, and this increases the likelihood of crack formation and breakage (Ndindeng et al. 2021). Cracked and broken grains are highly vulnerable to fungal infestation. Small-scale mechanical threshers (AfricaRice-SAED-ISRA (ASI)) adapted to the rice farm topology in SSA have been developed and are widely used in some countries such as Cote d’Ivoire and Senegal (Ogwuike et al. 2022). Rubber roll mills are recommended for rice milling over the Engelberg type mills. Rubber roll mills involve a multi-stage milling process of precleaning, husking, paddy separation, whitening, polishing, and grading, leading to a lower percentage of grain breakage.

**Fig. 1:**
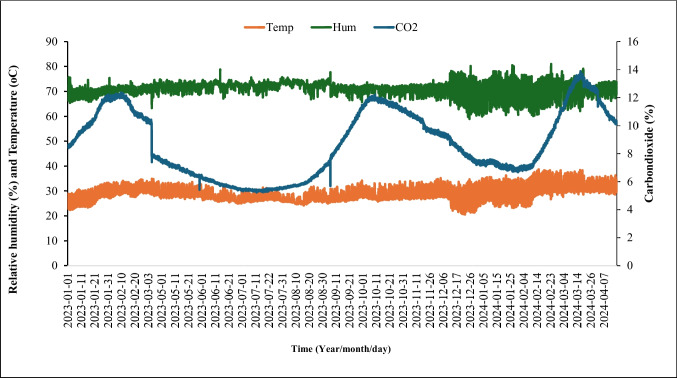
Temperature, relative humidity, and carbon dioxide levels in auto-CO_2_-generated anti-rodent hermetic storage cocoons installed in the semi-humid agroecological (Bouake) zone in Cote d’Ivoire

Rice seeds should be under conditions that do not favor fungal growth (e.g., in dry conditions, 8–10% moisture content), ideally in hermetic storage systems (airtight). Small-scale oxygen-proofed hermetic storage technologies have been developed to prevent rehydration from saturated ambient air or rain and to maintain temperature and relative humidity at levels that minimize microbial proliferation and seed quality deterioration. The principle of operation of these hermetic storage systems requires that rice grain be kept in an ERH that initially allows the metabolism of microorganisms and insects (that is, ERH < 85%) enclosed in the system for rapid reduction of oxygen and equivalent buildup of carbon dioxide levels. Hermetic storage technologies include Purdue Improved Crop Storage (PICS), GrainPro Superbags, and GrainPro cocoons (Chigoverah et al. 2018; Yewle et al. 2021). We conducted a pilot storage of rice seeds for twelve months in a hermetic cocoon and observed that the carbondioxide levels rapidly increased to 5.3% and remained above this level throughout the period, reaching a peak of 13.4% in the eleventh month of storage. Figure 1 presents the levels of CO_2_, relative humidity, and temperature in the hermetic storage cocoons for 12 months in the semi-humid agroecological zone (Bouake) in Cote d’Ivoire.

#### Capacity building for continuous surveillance of mycotoxins and consumer sensitization

Rice actors at all levels should be equipped with knowledge of the existence of mycotoxins, rapid detection methods, public health hazards in animals and humans, mitigation, and decontamination strategies. Specific modules for the capacity building of rice farmers, processors, and consumers should be aimed at lowering the risk of mycotoxin contamination of rice. In the case of rice farmers, capacity building should be centered on the selection of stress tolerant varieties (eliminating the likelihood of chalk formation and breakage during milling), the respect of good agronomic practices (dose respect and timely application of fertilizers, proper water, and weed management), harvesting at optimum maturity (minimize formation of fissures and breakage during milling), threshing with appropriate equipment (minimize breakage), and respect of adequate drying procedures. For the processors, capacity building should be focused on the adoption of processing methods that minimize grain breakage and improve quality, use of multistage rice mills, improved parboiling methods, and hermetic packaging. Consumers should be trained on appropriate handling (short-term storage of purchased milled rice in hermetic bags, selection of brands with high head rice and low chalkiness), and cooking methods (soaking of milled rice in hot water (leaching of water-soluble mycotoxins) and thorough washing before cooking. Finally, all rice actors should be continuously sensitized through education, seminars, workshops, and media on mycotoxin mitigation strategies.

The present study aimed to investigate the frequency of AFs in rice from markets in five SSA countries, model the contribution of chalkiness, grain shape, origin of grains, and processing type to AF concentration and total FBs, ZEN, and DON cooccurrence with total AFs. Domestic, imported, white, and parboiled rice showed detectable levels of total AFs, expressing cooccurrence with ZEN but not with total FBs and DON. The grain quality characteristic significantly affected AFs levels in rice. Slender, medium, and chalky grains expressed higher AFs than the corresponding nonchalky and bold grains. Of the 527 samples analyzed, total AFs occurred in 379 (3–3.9 µg/kg) and 180 were contaminated, with a concentration range of 4–89 µg/kg. Of the 50 highly contaminated AF samples purposefully selected for FBs, ZEN, and DON analysis, none was contaminated with, nor showed occurrence of FBs and DON, while there was cooccurrence of AFs with ZEN in 40% of the samples. Total AFs exceeded ML in 47.5% of the rice, while ZEN exceeded ML in 30% of the samples. These results underscore the need to concert efforts to ensure grain quality and to use the appropriate processing and handling technologies (processing, transportation, and packaging). Dry chain principles, hermetic storage packaging, and capacity building have been discussed as ecofriendly and affordable technologies to mitigate rice mycotoxin contamination by rice chain actors in SSA.

## Supplementary Information

Below is the link to the electronic supplementary material.

Supplementary file1 (DOCX 26 KB)

## Data Availability

The data that supports the findings of this study is provided within the manuscript and in the supplementary information table.

## Acronyms

AFB_1_: Aflatoxin B1
AFs: Aflatoxins
DON: Deoxynivalenol
FBs: Fumonisins
HRR: Head rice ratio
LWR: Length-to-width ratio
ML: Maximum level
SSA: Sub-Saharan Africa
ZEN: Zearalenone
