# Supplementary material for: Mycotoxin concentrations in rice are affected by chalkiness, grain shape, processing type, and grain origin
Source: Mycotoxin Res. 2024 Nov 27;41(1):163–77. doi: 10.1007/s12550-024-00575-w (PMC11757643; doi:10.1007/s12550-024-00575-w)
Supplement: Supplementary file 1 — Supplementary file1 (DOCX 26 KB) [file 12550_2024_575_MOESM1_ESM.docx]

Supplementary information

Supplementary file 1: Aflatoxin frequency in domestic and imported rice by grain shape and chalky status (µg/kg)

| Label | Domestic | |  | Imported | |  |
| --- | --- | --- | --- | --- | --- | --- |
|  | bold | medium | slender | bold | medium | slender |
| Rural | | | | | | |
| **Brown** | **/** | **6.23 (4.40 – 8.20)** | **/** | **/** | **/** | **/** |
| not chalky | / | 5.25 (4.40 – 6.10) | / | / | / | / |
| chalky |  | 8.20 | / | / | / | / |
| **White** | **3.65 (2.30 – 5.90)** | **3.56 (1.80 – 7.30)** | **4.81 (2.30 – 14.50)** | **3.73 (3.00 – 5.10)** | **3.90 (0.99 – 15.10)** | **11.36 (3.30 – 75.90)** |
| not chalky | 3.53 (2.30 – 5.90) | 3.38 (1.80 – 5.70) | 7.63 (3.90 – 14.50) | 3.73 (3.00 – 5.10) | 3.99 (0.00 – 15.10) | 3.98 (3.30 – 4.70) |
| chalky | 4.0 | 4.14 (2.10 – 7.30) | 3.75 (2.30 – 5.10) | / | 3.69 (2.20 – 4.10) | 15.59 (3.40 – 75.90) |
| **Parboiled** | **/** | **/** | **3.25 (2.60 – 3.90)** | **/** | **2.75 (2.40 – 3.10)** | **2.77 (1.20 – 4.0)** |
| not chalky | / | / | 3.25 (2.60 – 3.90) | / | 3.10 | 2.76 (1.20 4.0) |
| chalky | / | / | / | / | 2.40 | 3.0 |
| overall-shape | 3.65 (2.30 – 5.90) | 4.90 (1.80 – 8.20) | 4.03 (2.30 – 14.50) | 3.73 (3.00 – 5.10) | 3.33 (0.99 – 15.10) | 7.07 (1.20 – 75.90) |
| Overall-origin | 4.43 (1.80 – 14.50) | | | 4.31 (0.99 – 75.90) | | |
| Overall-market type | 4.44 (0.99 – 75.90) | | | | | |
| Urban | | | | | | |
| **Brown** | **3.80** | **4.40 (1.50 9.00)** | **3.40 (2.80 – 3.90)** | **/** | **3.98 (3.00 – 4.90)** | **5.25 (4.70 – 5.80)** |
| not chalky | 3.80 | 4.81 (2.30 – 9.00) | 3.60 (3.20 – 3.90) | / | 3.98 (3.00 – 4.90) | 5.25 (4.70 – 5.80) |
| chalky | / | 1.50 | 2.80 | / | / | / |
| **White** | **3.61 (2.10 – 6.00)** | **4.92 (1.60 – 89.80)** | **5.63 (2.10 – 29.00)** | **3.34 (1.80 – 4.50)** | **3.70 (1.40 – 13.60)** | **4.36 (2.00 – 22.60)** |
| not chalky | 3.60 (2.10 – 6.0) | 3.57 (1.60 – 8.00) | 5.82 (2.10 – 29.00) | 3.34 (1.80 – 4.50) | 3.50 (1.40 – 13.60) | 4.30 (2.00 – 22.60) |
| chalky | 3.70 | 6.55 (1.60 – 89.80) | 5.49 (2.80 – 23.90) | / | 3.93 (2.30 – 11.80) | 4.43 (2.10 – 11.40) |
| **Parboiled** | **/** | **4.00 (3.50 – 4.50)** | **3.69 (2.70 – 5.10)** | **/** | **3.38 (2.10 – 4.40)** | **3.33 (1.30 – 5.60)** |
| not chalky | / | 4.00 (3.50 – 4.50) | 3.63 (2.70 – 5.10) | / | 3.38 (2.10 – 4.40) | 3.33 (1.30 – 5.60) |
| chalky |  | / | 4.10 | / | / | / |
| overall-shape | 3.71 (2.10 – 6.00) | 4.44 (1.50 – 89.80) | 4.24 (2.10 – 29.00) | 3.34 (1.80 – 4.50) | 3.67 (1.40 – 13.60) | 4.31 (1.30 – 22.60) |
| Overall-origin | 4.28 (1.50 – 89.80) | | | 3.88 (1.30 – 22.60) | | |
| Overall-market type | 4.31 (1.30 – 89.80) | | | | | |

Values are the mean (min – max) of aflatoxin concentration in each group.
